# Community Member and Stakeholder Perspectives on a Healthy Environment Initiative in North Carolina

**DOI:** 10.5888/pcd12.140595

**Published:** 2015-08-13

**Authors:** Lori Carter-Edwards, Abby Lowe-Wilson, Mary Sherwyn Mouw, Janet Yewon Jeon, Ceola Ross Baber, Maihan B. Vu, Monique Bethell

**Affiliations:** Author Affiliations: Abby Lowe-Wilson, UNC Center for Health Promotion and Disease Prevention, University of North Carolina at Chapel Hill, Chapel Hill, North Carolina; Mary Sherwyn Mouw, Cancer Control Education Program, Lineberger Cancer Center, University of North Carolina at Chapel Hill, Chapel Hill, North Carolina; Janet Yewon Jeon, Department of Health Behavior, Gillings School of Global Public Health, University of North Carolina at Chapel Hill, Chapel Hill, North Carolina; Ceola Ross Baber, Department of Leadership Studies, North Carolina Agricultural and Technological State University, Greensboro, North Carolina; Maihan B. Vu, Department of Health Behavior, Gillings School of Global Public Health, and UNC Center for Health Promotion and Disease Prevention, University of North Carolina at Chapel Hill, Chapel Hill, North Carolina; Monique Bethell, Department of Public Health Education, North Carolina Central University, Durham, North Carolina, and Chronic Disease and Injury Section, North Carolina Division of Public Health, Raleigh, North Carolina.

## Abstract

**Introduction:**

The North Carolina Community Transformation Grant Project (NC-CTG) aimed to implement policy, system, and environmental strategies to promote healthy eating, active living, tobacco-free living, and clinical and community preventive services to advance health equity and reduce health disparities for the state’s most vulnerable communities. This article presents findings from the Health Equity Collaborative Evaluation and Implementation Project, which assessed community and stakeholder perceptions of health equity for 3 NC-CTG strategies: farmers markets, shared use, and smoke-free multiunit housing.

**Methods:**

In a triangulated qualitative evaluation, 6 photo elicitation (PE) sessions among 45 community members in 1 urban and 3 rural counties and key informant interviews among 22 stakeholders were conducted. Nine participants from the PE sessions and key informant interviews in the urban county subsequently participated in a stakeholder power analysis and mapping session (SPA) to discuss and identify people and organizations in their community perceived to be influential in addressing health equity–related issues.

**Results:**

Evaluations of the PE sessions and key informant interviews indicated that access (convenience, cost, safety, and awareness of products and services) and community fit (community-defined quality, safety, values, and norms) were important constructs across the strategies. The SPA identified specific community- and faith-based organizations, health care organizations, and local government agencies as key stakeholders for future efforts.

**Conclusions:**

Both community fit and access are essential constructs for promoting health equity. Findings demonstrate the feasibility of and need for formative research that engages community members and local stakeholders to shape context-specific, culturally relevant health promotion strategies.

## Introduction

Chronic diseases in North Carolina account for nearly 60% of all deaths in the state (1,2). Modifiable burdens of these diseases are often greater in rural counties or counties with high rates of poverty, poor access to health care, and high proportions of people of color (2). Effective interventions are needed at the institutional, organizational, system, and policy levels for sustainable change, especially among groups that are disproportionately affected by health disparities (3). To design and implement such interventions, it is important to understand the issues and perspectives of community members (4).

The Health Equity Collaborative Evaluation Planning and Implementation Project (HECEPP) was one of 4 evaluation efforts of the North Carolina Community Transformation Grant Project (NC-CTG), a statewide initiative designed to promote healthier environments through local implementation of evidence-based strategies. HECEPP, the only qualitative evaluation effort of NC-CTG, was conducted to investigate perspectives of health equity (ie, all residents having access to opportunities for optimal health) in rural and urban counties in 2 NC-CTG regions. 

Using a unique, triangulated evaluation approach, we assessed community member and stakeholder perceptions of 3 NC-CTG strategies: farmers markets (healthy eating), shared use (active living), and smoke-free multiunit housing (tobacco-free living). The primary evaluation question was: How do residents from urban and rural counties perceive health equity in terms of farmers markets, shared use agreements, and smoke-free policies in multiunit housing? The primary hypothesis was that there would be differences in health equity perceptions between urban and rural county residents. This article presents the overall findings from this evaluation.

## Methods

To improve health and prevent chronic diseases at the community level, the national CTG Program provided implementation and capacity-building support to awardees across the United States. Intervention areas included healthy eating, active living, tobacco-free living, and clinical and community preventive services to prevent and control high blood pressure and high cholesterol. The CTG was funded by the Affordable Care Act’s Prevention and Public Health Fund, through the Centers for Disease Control and Prevention. NC-CTG was awarded to the state’s Division of Public Health Chronic Disease and Injury Section (DPH-CDI), which worked with state and local partners to implement evidence-based strategies to support healthier environments. Strategies were to adhere to CTG principles: maximizing health impact through prevention, using and expanding the evidence base, and advancing health equity. Although NC-CTG was originally designed to be implemented from 2012 through 2016, the project concluded in September 2014 because of CTG federal budget cuts (5).

DPH-CDI contracted with the University of North Carolina at Chapel Hill to evaluate perceptions of health equity in the context of CTG strategy implementation. With community engagement at its core, HECEPP staff developed a comprehensive evaluation framework to guide collaboration with NC-CTG state staff and the HECEPP Advisory Group, consisting of community and academic leaders with expertise in community engagement and health promotion. The framework included a communication plan for sharing information with DPH-CDI and the strategy-specific evaluation teams (6), orientation of the evaluation teams to culturally competent evaluation readiness (at the request of NC-CTG state staff) (7), and a health equity evaluation plan (8). The evaluation plan included 3 qualitative approaches: photo elicitation (PE) sessions; key informant interviews; and stakeholder power analysis and mapping (SPA), a group process of identifying and mapping stakeholder influence and issue-specific interest (9–12). The approach was designed to evaluate health equity perceptions across all 3 strategies in 1 urban county and 1 strategy each in 3 rural counties (year 1), across all 3 strategies in 1 rural county and 1 strategy each in 3 urban counties (year 2), and collectively across all participating rural and urban counties (year 3). The evaluation for year 1 was successfully completed by September 2014. This evaluation included 1 urban county (Gaston) and 3 rural counties (Lee, farmers markets; Scotland, shared use; and Montgomery, smoke-free multiunit housing). PE was the primary qualitative evaluation approach (per the requirements of NC-CTG), and key informant interviews and SPA were used as complementary approaches.

PE participants were recruited through 5 HECEPP-trained community coordinators working in the 4 participating counties. The PE target sample was 60 community members (6 sessions, 10 per session) residing in 1 of 4 participating counties. Key informants were recruited by HECEPP staff from a stakeholder list of NC-CTG county and regional-level staff and community leaders and partners in the 2 participating NC-CTG regions. The key informant interview target sample was 24 stakeholders residing or working in one of the participating counties. The original SPA target sample was 30 people (2 sessions, 15 per session) across the 4 counties who also participated in PE sessions or as a key informant. Because of funding cuts, SPA was conducted only in Gaston County.

For PE, community members attended an informational meeting, completed an informed consent and demographic survey, and received a disposable camera (used and subsequently mailed to HECEPP staff). Approximately 3 weeks later, they returned to review their photos and collectively select, describe, and title the 5 photos they felt best reflected health equity issues in their community. They responded to focus group questions on experiences related to the selected photos, including people, places, and things perceived to have the greatest impact on health disparities and health equity in their communities and what can maximize health equity. Key informants each signed an informed consent and demographic survey, and participated in a 30- to 40-minute phone interview. They discussed their connection to NC-CTG; perceptions on health equity, health disparities, and community engagement; and the progress of and lessons learned from NC-CTG. For SPA, community members and stakeholders recruited from Gaston County PE sessions and key informant interviews each completed a power relationship “wheel,” a visual tool for identifying influential relationships they have with stakeholders in their community; and they collectively mapped these stakeholders as having high or low interest and power in addressing health equity surrounding NC-CTG strategies. All sessions were audio-recorded and transcribed.

For this evaluation, PE data were treated as the reference, and key informant interview and SPA data were compared with PE findings. Separate codebooks were created to analyze data from PE sessions and key informant interviews. Topical codes were drawn from interview guides (eg, influential people and organizations) and participants’ words, (eg, convenience, cost, safety); inductive codes emerged on re-reading (eg, values, community fit). For PE analysis, HECEPP staff developed the codebook iteratively, focusing on what participants said about each strategy specifically and what they described collectively as influencing health equity. To ensure intercoder reliability, HECEPP staff worked together to apply codes to 2 transcripts and compared others that were coded independently, discussing discrepancies before individually completing coding. Data were analyzed using ATLAS.ti version 7.1.8 (Scientific Software Development GmbH). This project was approved by the institutional review board at the University of North Carolina at Chapel Hill.

## Results

PE participants had a mean age of 50.7 years and had lived in their counties approximately 36 years (Table 1). Approximately 77% of participants were female, 80% were black, and slightly more than one-third had achieved a high school education or less. Mean age of the 22 key informants was 45.7 years; 76% of key informants were female, 73% were white, 46% had at least an associate’s degree, and they lived or worked in their county roughly 18 years. Nine of the 30 PE and key informant interview participants also participated in SPA.

**Table 1:** Participant Demographics of Photo Elicitation Sessions and Key Informant Interviews, by County Urbanity or Rurality, North Carolina Community Transformation Grant Project, 2014

Characteristic	Total Sample	Farmers Markets		Shared Use			Smoke-Free Multiunit Housing	
		Urban^a^	Rural^b^	Urban^a^	Rural^c^^,^^d^		Urban^a^	Rural^e^^,^^f^
**Photo Elicitation Sessions**								
**No. of participants**	45	5	4	8	10		8	10
**Mean age, y (SD)**	50.7 (16.6)	54.2 (12.1)	47.8 (12.3)	45.4 (22.5)	54.8 (16.4)		55.8 (14.7)	46.1 (17.5)
**Sex, % (n = 44)**								
Female	77.3	80.0	50.0	87.5	77.8		75.0	80.0
Male	22.7	20.0	50.0	12.5	22.2		25.0	20.0
**Race, %**								
Black	80.0	80.0	75.0	75.0	80.0		75.0	90.0
White	15.5	20.0	25.0	0.0	20.0		25.0	10.0
Other	4.5	0.0	0.0	25.0	0.0		0.0	0.0
**Education, %**								
High school	37.8	60.0	0.0	37.5	30.0		37.5	50.0
Some college/trade	22.2	0.0	25.0	12.5	20.0		12.5	50.0
College degree	40.0	40.0	75.0	50.0	50.0		50.0	0.0
**Mean time living in county, y**	35.7	32.2	32.5	30.3	33.7		43.4	38.8
**Perceived influence/power in county, % (n = 44)**								
Yes, where I work	40.9	40.0	50.0	50.0	40.0		25.0	44.4
Yes, where I live	56.8	60.0	75.0	75.0	80.0		25.0	33.3
**Key Informant Interviews**								
**Characteristic**	**Total Sample**	**Urban^a^^,^^d^**				**Rural^b^^,^^c^^,^^e^**		
**No. of participants**	22	9				13		
**Mean age, y (SD)**	45.7 (14.5)	39.7 (14.3)				49.9 (13.7)		
**Sex, % (n = 21)**								
Female	76.2	87.5				69.2		
Male	23.8	12.5				30.8		
**Race, %**								
Black	27.3	22.2				30.8		
White	72.7	77.8				69.2		
**Education, %**								
Associate/bachelor degree	45.5	44.4				46.2		
Master’s degree	45.5	45.5				38.5		
Doctorate/professional	9.1	0.0				15.4		
**Mean time living/working in county, y**	17.6	13.1				20.7		
**Perceived influence/power in county, %**								
Yes, where I work	68.2	77.8				61.5		
Yes, where I live	54.5	55.6				53.8		

Abbreviation: SD, standard deviation.

a Gaston County.

b Lee County.

c Scotland County.

d Sex was missing for 1 participant.

e Montgomery County.

f Perceived influence/power missing for 1 participant.

Overall, community fit and community access were important health equity issues for community members and stakeholders. No distinct differences in perceptions were observed between the counties.

### Photo elicitation findings

Multiple factors influenced PE participants’ perspectives on the 3 NC-CTG strategies (Table 2). Both rural and urban participants individually discussed safety, cost, quality, convenience, values, perceived norms, and awareness. When viewed collectively, 2 broader community-level themes emerged: access and community fit. Access referred to how easy or difficult it was for groups or communities to be connected to or use resources related to a NC-CTG strategy. Community fit was the collective acceptability or desirability of a strategy for community members. If an implemented strategy was deemed inconvenient for many respondents, it was viewed as an access issue. Likewise, community fit was shaped by commonly shared, individual-level factors such as values, perceived norms, knowledge, and safety. Unequal levels of access or community fit influenced the perceived health equity impact of a strategy (Figure).

**Table 2:** Health Equity Factors That Urban and Rural Community Members Found Important, by Strategy, North Carolina Community Transformation Grant Project, 2014

Health Equity Factor^a^	Farmers Markets	Shared Use	Smoke-Free Multiunit Housing
Awareness	X	X	X
Convenience	X	X	
Cost	X		
Perceived norms			X
Quality	X	X	
Safety		X	X
Values	X		X

a An “X” denotes that the factor emerged as a key theme among participants for the specified strategy.

**Figure:**
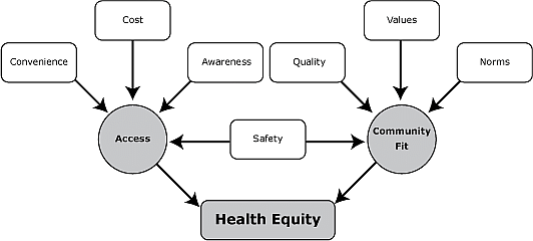
Conceptual model of health equity through contextual perceptions of community members and other stakeholders.

In the PE sessions, there was little direct reference to racial groups’ use of the strategies. Rather, PE participants focused on how each strategy was relevant for them individually and how the strategies pertained to groups defined by characteristics other than race. For example, participants were keenly interested in how the strategies might affect groups perceived as vulnerable: the elderly, the disabled, the poor, children and young parents, smokers and nonsmokers, farmers, and working people. 

Participants discussed location of farmers markets, produce quality, transportation, acceptable use of Electronic Benefit Transfer (EBT) cards, and benefits to farmers and the local economy. Convenience stood out as a particular concern, as reflected in this exchange between 2 Gaston County participants:

Taking a closer look at the times, the times did not suit me.

You got a life, right?

Well, I work.

Thus, working people as a group cannot access the markets. Location and safety were the main concerns about shared-use facilities. For example, a Gaston County participant was concerned about traffic:

The children . . . have to cross the road. . . . It’s not safe; they’ll run across.

When discussing a photo of a walking trail, 2 Scotland County residents reflected,

I think it started getting dark. I might not want to be out there . . .

I think it is in a positive, safe environment. . . . So I think at night, it would be safe. But for a woman to go by herself, I would not advise it, because there is not enough light.

Therefore, safety was considered a limit to shared-use access for women and children.

Attitudes toward smoke-free policies in multiunit housing were shaped by health concerns (personal health and experiences with others’ tobacco-related illnesses), worries about fire hazards, and considerations about whether such policies were fair to smokers. A Montgomery County resident expressed concerns, for example, when smoke-free policies were seen to force elderly smokers to stand outside in inclement weather:

It came, that rain and sleet . . . they had to stand out in the elements. . . . Some were wrapped in quilts, some were wrapped in coats. . . . These are older people, I would say from 65 to 80. . . . You really get a guilt trip going.

This remark reflected a negative aspect of the community fit strategy, because the strategy conflicted with commonly shared values about respecting and protecting elders.

PE participants made practical suggestions to increase the impact of the strategies; examples cited were providing transportation to, expanding the hours of, and advertising farmers markets; improving existing spaces for physical activity and increasing their visibility; including both smokers and nonsmokers in implementing smoke-free policies in multiunit housing properties; and incorporating safety issues into these discussions.

### Findings of key informant interviews and SPA

As did PE participants, key informants emphasized access. Key informants’ work with CTG strategies often addressed the same factors influencing access as those described by PE participants. They focused on cost and convenience, for example, in situating a new farmers market adjacent to a federally qualified health center, and in efforts to expand methods of payment to include EBT cards. Key informants who were county or regional NC-CTG staff implemented smoke-free policies in properties designated for people with disabilities, mental illness, and substance use, improving these vulnerable groups’ access to clear air. In contrast to PE participants, however, few key informants referenced community fit of the proposed strategies. Most of what the key informants and SPA participants said about community pertained to partnerships with policy makers and organizations. The common thread in the key informant interviews and SPA was that the power to make systems and environmental changes comes from multisector partnerships. When discussing community engagement, key informants generally described building relationships with partners who could successfully implement strategies. This concern reflected resourcing, in that the CTG often provided funding or technical assistance for projects conducted by other entities, including government agencies (eg, school districts, cooperative extensions), faith communities, property managers, farmers, hospitals, city planners, and nongovernment organizations. Key informants and SPA participants deemed multisector partnerships essential to NC-CTG’s successes.

Key informants and SPA participants stressed the difficulties of engaging local policy makers, and acknowledged policy makers’ competing responsibilities (Table 3). In the case of smoke-free policies, the historical significance of tobacco in local economies was seen as a barrier, as were assumptions about the impact of smoke-free policies on farmers. Safety concerns (eg, drug dealers loitering in public spaces), and opposition to tax increases (eg, funding facility upgrades) were seen as barriers to policy makers’ supporting shared-use agreements. Some participants thought the CTG being funded by the Patient Protection and Affordable Care Act deterred some elected officials they believed might have otherwise expressed support.

**Table 3:** Stakeholder Power Analysis Mapping, by Strategy, North Carolina Community Transformation Grant Project, 2014

Group	Strategy		
	Farmers Markets	Shared Use	Smoke-Free Multiunit Housing
**High interest and high power**	Foundations (eg, United Way, YMCA) Health care commissioners Local service organizations Supportive county commissioners	Foundations (eg, United Way, YMCA) Health care commissioners Pastors School principals School board members Parks and recreation Programs promoting physical activity (eg, Girls on the Run) Supportive city council members Supportive county commissioners	Foundations (eg, United Way, YMCA) Health care commissioners Pastors School principals School board members Local hospital Local service organizations (eg, crisis pregnancy group to stop smoking during pregnancy)
**High interest and low power**	Farmers	Sports organization (eg, Amateur Athletic Union local chapter)	NA
**Low interest and high power**	Nonsupporting county commissioners Farmers market board	Nonsupporting county commissioners Drug dealers	Nonsupporting county commissioners Smokers
**Low interest and low power**	Tenants with limited mobility Low-income tenants	Law enforcement	NA

Abbreviation: NA, not applicable.

Key informants were more likely to raise the issue of race than were the PE participants. Many key informants felt there had not been enough grassroots participation in NC-CTG projects overall, but few offered an explanation. One key informant was critical of there not being any community members on NC-CTG’s regional planning team; another described the need for formative evaluation with communities:

If you don’t get out with your population, and I am talking about every aspect of it . . . you can assume they need help, but you don’t know what they need help with.

Another explained that the process of engaging community members takes time:

We decided to take it slow, because we wanted to make sure we were spending money where it was needed . . . to have that health equity focus . . . trying to figure . . . that people actually want these things. . . . If you're going to make that community-level change, you’re going to have to [engage] with and through people, and that starts again with the relationships.

These insights centered on community engagement and community fit. When partner organizations were rooted in community, projects were described as well-received and smoothly implemented, as when NC-CTG staff worked with an established community–university partnership to conduct a survey of local farmers. Several key informants mentioned a successful meeting between NC-CTG staff and multiunit housing residents about smoke-free policies. Key informants were most confident of their impact on promoting health equity where staff had been most directly engaged with the people being affected.

An NC-CTG administrator pointed out that, although project leadership was tasked with promoting health equity, partners from other sectors may have different interests and priorities. Key informants and PE participants offered cautionary insights into how partner organizations can influence health equity. While taking photos, a PE participant noticed that a management company had implemented smoke-free policies differently at 2 properties. One had an outdoor smoking shelter; the other did not. The participant saw disparity in treatment of the 2 groups of residents, thus varying access to clean air. The inferred questions were: Who was differentially affected by the policies? Why was the policy implemented in that way? Was there something different about the groups that led to the different rules?

## Discussion

HECEPP used a comprehensive qualitative approach to gain an overview of contextual conditions and social and cultural relevance of the 3 NC-CTG strategies. Findings indicate that community fit and access are important considerations for addressing health equity. Missing was a sense that there was an overarching, shared vision of how NC-CTG policy, structural, and environmental changes were to reduce health disparities and for which groups. The conceptual model derived from the PE findings (Figure) may be useful in understanding how such interventions can work and in creating measures of community fit for evaluating short-term outcomes. Identifying measurable mediators that reflect community fit will strengthen the evidence base for strategies whose effects on health outcomes and health disparities are likely to be realized in the long term (13).

One limitation of the evaluation is that there was minimal mention of race or racism as a health equity construct in this project. Although key informants in the CTG administrative roles talked about race more often than did other stakeholders, there were missed opportunities in the PE sessions and SPA to further probe relevant comments (eg, the potential influence of faith communities in the CTG projects, references to perceived unequal treatment). Most key informants were white and had higher education (Table 1). In addition to completing urban and rural health equity comparisons, future work must explore how race interacts with other social determinants to influence population health. Although NC-CTG ended early, these findings should be a springboard to additional work addressing the strategies in counties involved in this project.

Recognizing the importance of sharing results to provide community members and stakeholders with relevant, accessible, actionable information, HECEPP findings have been presented at a Gaston County Town Hall meeting, where some attendees were stakeholders identified through SPA, including a local official, a health care system representative, community leaders and members, and NC-CTG local and state staff. Unintended positive consequences of this evaluation were the subsequent presentations of HECEPP findings at the Gaston County Board of Health, and the DPH-CDI Section Health Equity Workgroup meetings. In fact, the DPH-CDI Section Health Equity Workgroup has used the HECEPP framework to explore its efforts to address health equity.

This study contributes to the literature by exploring methods to engage community members in the evaluation process. It identifies important considerations for structural, environmental, and policy health promotion initiatives, particularly the need for formative research with communities about their interests and perceived needs. PE participants’ practical suggestions and context-specific ideas have potential for grassroots participation and community-driven initiatives for promoting healthy eating, physical activity, and tobacco use cessation. Participants’ future efforts can involve people in their respective counties and catalyze a more community-engaged process for future policy and systems initiatives.
